# Ruminal Crude Protein Degradation Determined in Sacco and by Co-Incubation of *Streptomyces griseus* Protease and Carbohydrases

**DOI:** 10.3390/ani14202982

**Published:** 2024-10-16

**Authors:** Paul Okon, Monika Wensch-Dorendorf, Martin Bachmann, Dirk von Soosten, Ulrich Meyer, Jörg-Michael Greef, Sven Dänicke, Annette Zeyner

**Affiliations:** 1 Institute of Agricultural and Nutritional Sciences, Martin Luther University Halle-Wittenberg, 06120 Halle (Saale), Germany; 2 Friedrich-Loeffler-Institut (FLI), Federal Research Institute for Animal Health, Institute of Animal Nutrition, 38116 Braunschweig, Germany; 3 Julius Kühn-Institut, Federal Research Centre for Cultivated Plants, Institute for Crop and Soil Science, 38116 Braunschweig, Germany

**Keywords:** protein evaluation, in vitro method, α-amylase, Viscozym^®^ L, simultaneous incubation, antibiotic solution

## Abstract

**Simple Summary:**

Ruminal protein degradation of seven feedstuffs was estimated in sacco and in vitro by using a protease assay. In sacco protein degradation data were used as reference. The accuracy of the protease assay is reduced by two methodological aspects. During in vitro incubation, microbial activity induced by microbial colonization of the feedstuff may increase, requiring antibiotic solutions in protease assays. The protease alone cannot sufficiently hydrolyze protein–carbohydrate complexes. Therefore, a carbohydrase of fiber- or starch-hydrolyzing activity was added to the protease assay as a simultaneous incubation. The antibiotic solution reduced protein degradation by protease. The antibiotic solution is recommended to prevent microbial activity and improve standardization of the protease assay. Differences between in sacco and in vitro degradation data were not essentially reduced by additional carbohydrases. Unfavorable incubation conditions and the inhibitory effect of protease on the carbohydrase activity during simultaneous incubation may be responsible for the insufficient hydrolysis of protein–carbohydrate complexes by the carbohydrases. It does not seem promising to incubate protease and carbohydrase simultaneously.

**Abstract:**

The objectives of the study were to examine the effect of an antibiotic solution applied in the *Streptomyces griseus* protease method (SGPM) and the effect of carbohydrases in SGPM on the effective crude protein (CP) degradation (ED) with reference to in sacco ED. For this purpose, the ruminal CP degradation of rapeseed meal, dried distillers’ grains with solubles, wheat grain, corn grain, corn silage, grass silage and partial crop field pea silage was determined in sacco using three rumen-fistulated dairy cows and in vitro using SGPM. The impact of the antibiotic solution on CP degradation by *S. griseus* protease was investigated by supplementing SGPM with Penicillin–Streptomycin solution to reduce microbial mass proliferation during incubation. The carbohydrase α-amylase or Viscozym^®^ L (cell wall-degrading enzyme mixture) was added to the SGPM at four different doses simultaneously as a co-incubation to improve feed protein accessibility. For most feedstuffs, ED was lower when the antibiotic solution was used in SGPM (*p* < 0.05). The use of an antibiotic solution in the SGPM is recommended to standardize the SGPM. The in sacco ED values were significantly underestimated by the SGPM and by the SGPM with co-incubated carbohydrase (*p* < 0.05). Co-incubation of *S. griseus* protease and carbohydrase was not successful in reducing the differences to the in sacco CP degradation.

## 1. Introduction

In ruminants, the key to efficient use of feed protein is to know its rate of ruminal protein degradation [1]. Various approaches to determine the ruminal crude protein (CP) degradation rely on the use of animals or laboratory methods. The real reference for measuring ruminal CP degradation is the in vivo method as ruminal CP degradation, microbial contamination and endogenous proteins are measured in the same animal [2]. The complexity of the in vivo method and the bias of the results caused by markers make it difficult to routinely determine ruminal CP degradation [1,3]. An accepted reference method is the in sacco determination of CP degradation using rumen-fistulated cows [4]. This method is standardized, but time-consuming and labor-intensive. Furthermore, factors such as diet, animal species and the microbial contamination of incubated feeds generate large variation in the measured data [1,5,6]. Alternative methods for estimating ruminal CP degradation that do not include animals, such as the purely enzymatic *Streptomyces griseus* protease method (SGPM) according to Licitra et al. [7], appear to be promising approaches. Challenges in terms of time, cost, ethics and logistics are reduced and, thus, the standardization of these in vitro methods seems to be more achievable. However, the accuracy of the SGPM may be limited by two methodological shortcomings.

In a previous study [8], we used SGPM in 40 feedstuffs for which in sacco degradation data were available. The in sacco CP degradation data were underestimated by SGPM in most of the feedstuffs. This was probably related to the feed-specific matrices of proteins and carbohydrates. Within such a matrix, the protein is bonded and less accessible to the *Streptomyces griseus* protease (SGP). The addition of a carbohydrase with amylolytic and fibrolytic activity to the SGPM appears to be a suitable approach to better hydrolyze the protein–carbohydrate matrices [8]. The carbohydrases Termamyl 2X^®^ (α-amylase) and Viscozym^®^ L (a mixture of cell wall-degrading enzymes such as cellulases, hemicellulases, pectinase and ß-glucanase) were used to improve the degradation of CP by SGP as a pre-incubation step [9]. However, pre-incubation requires additional pH adjustment, centrifugation, decantation and rinsing, which are potential sources of error and are time-consuming [10]. Recently published data have shown that the co-incubation of a carbohydrase (α-amylase/Viscozym^®^ L) and SGP is possible at incubation conditions set by the SGPM (pH 6.75, 39 °C), although these are not optimal for each of the enzymes [11]. However, adverse effect of the SGP were also evident as SGP seemed to reduce the activity of α-amylase and Viscozym^®^ L [11]. The effect of SGP during co-incubation on α-amylase/Viscozym^®^ L might be compensated for by increasing the doses of the carbohydrases. Further investigations are necessary to prove this hypothesis with reference to in sacco CP degradation data.

The second point concerns the increase in microbial activity during the co-incubation of SGP and carbohydrase favored by the release of degradable nutrients [12]. Because of this, the use of antibiotics is required to reduce microbial activity, other than that suggested in the original SGPM protocol [7]. The combined preparation of Penicillin–Streptomycin appears to be suitable for this purpose, as it is effective against gram-positive and gram-negative bacteria and has been used with trypsin in cell cultures [13]. The use of a Penicillin–Streptomycin solution in SGPM should be investigated first to ensure that there are no adverse effects on CP degradation by SGP.

We hypothesized that an additional Penicillin–Streptomycin solution should have no effect or just a marginal effect on CP degradation, as it has been used with trypsin.

Additional carbohydrases assist the SGP to hydrolyze proteins from the matrix and reduce the difference to the in sacco ED reference.

The objectives of this study were to investigate the impact of a Penicillin–Streptomycin solution applied to the SGPM and the effect of α-amylase or Viscozym^®^ L on effective CP degradation (ED) in the SGPM with reference to in sacco ED.

## 2. Materials and Methods

### 2.1. Feedstuffs

The selection of feedstuffs for this study was on the basis of the results of a previous study in which the ED was estimated in sacco and by using SGPM [8]. The differences between in sacco and SGPM-estimated ED were clustered. One feedstuff was selected from each cluster, representing all feedstuffs of the respective cluster in terms of nutrient composition and treatment. In total, the following seven feedstuffs were used: rapeseed meal, wheat grains, dried distillers’ grains with solubles (DDGS), corn grains, corn silage, grass silage and partial crop field pea silage (PCFPS).

The rapeseed meal was provided by Raiffeisen Waren GmbH (Kassel, Germany). Wheat grains and DDGS were provided by producers who want to remain anonymous. The wheat represented the major component of DDGS for which the producer did not provide us any information. The corn variety LG 30.258 was grown and harvested as grain in November 2021 on the experimental fields near the Institute of Animal Nutrition of the Friedrich-Loeffler-Institut (FLI) in Braunschweig (Germany).

The corn silage (corn variety LG 30.258) and grass silage plant materials were grown and harvested on adjacent fields of the FLI in Braunschweig (Germany). At the time of harvest, the corn plants ranged in Biologische Bundesanstalt für Land- und Forstwirtschaft, Bundessortenamt und Chemische Industrie growth stages between 82 and 85 [14]. The corn plant material was harvested as a whole crop, chopped to a particle length of 8–10 mm and ensiled in silo stock in September 2021. The grass plant material was harvested as a second cut in June 2020, chopped to a particle length of 20–30 mm and ensiled in silo stock and opened in April 2021. The pea variety *Astronaute* (Norddeutsche Pflanzenzucht Hans-Georg Lembke KG, Holtsee, Germany) was sown in April/May 2020 near the Saxon State Farm for Teaching and Research Köllitsch and harvested in July 2020 by direct cutting of the plants at an approximate height of 25 cm. Subsequently, the material was ensiled in round bales which were opened in the period from November 2020 to January 2021. Aliquot samples of the round bales were merged into one bulk sample. Pea harvesting and processing are described in detail by Okon et al. [15]. All silages were ensiled without silage additives.

### 2.2. In Sacco Procedure

The in sacco experiment was conducted in compliance with German animal protection laws and approved by the Lower Saxony State Office for Consumer Protection and Food Safety (approval no. 33.19-42502-04-17/2577), in consultation with an independent ethics committee.

The in sacco experiment was conducted in 2021 and 2022 according to Wroblewitz et al. [16] at the FLI experimental station in Braunschweig (Germany) using three lactating German Holstein dairy cows fitted with permanent cannulas in the dorsal rumen. The average body weight was 691 ± 56 kg in 2021 and 632 ± 84 kg in 2022; note that different cows were used in 2022. The days in milk of the cows ranged between 179 and 284 (4th–6th lactation) in 2021 and between 123 and 203 (2nd–5th lactation) in 2022. The average milk yields were 30 ± 9 kg and 34 ± 7 kg, respectively. The cows had free access to tap water and were fed *ad libitum*. The partial mixed ration (PMR) was formulated according to the Society of Nutrition Physiology recommendations [17] and consisted of a dry matter (DM) basis of 50% corn and 50% grass silage in 2021 and of 57% corn, 29% grass silage and 14% concentrate (33% wheat, 29% dried sugar beet pulp, 16% rapeseed meal, 18% soybean meal, 2.5% minerals, 1.5% soybean oil and 1.3% urea) in 2022. The average fresh matter intake was 35 ± 7 kg in 2021 and 37 ± 6 kg in 2022. Additionally, the cows had access to an automatic concentrate feeder, which provided a maximum of 4 ± 2 kg/d in 2021 and 8 ± 2 kg/d in 2022.

The feedstuffs for ruminal incubation were ground through a 3 mm sieve (Retsch ZM 100, Haan, Germany), and amounts of 4 g were weighed into pre-washed and dried nylon bags (100 × 200 mm; pore size: 50 ± 10 µm; Ankom Technology, New York, NY, USA), closed with a cable tie and fixed to a cast iron ring (542 g) with an additional weight (913 g). Before incubation, the bags were placed in warm tap water for at least one minute. The nylon bags were incubated according to Paine et al. [18] as a complete exchange. Each incubation time point was incubated separately by removing all bags from the rumen at the end of the incubation period and placed into ice water to stop microbial activity. The time series comprised seven separate incubation time points (2, 4, 8, 16, 24, 48 and 72 h). Subsequently after ruminal incubation, the bags were rinsed with cold tap water to remove rumen digesta, washed in a washing machine (Gorenje, WA 1042, Velenje, Slovenia) without spinning for 20 min with cold tap water and dried at 60 °C for 24 h. After cooling down in an exicator, the bags were weighed and all the residues of one animal were pooled into one bulk sample. The nylon bags of incubation time 0 h were washed in the washing machine and prepared for analysis as described above without ruminal incubation. Then, the pooled samples were analyzed for DM and CP concentration.

### 2.3. In Vitro Procedure

The SGPM was conducted according to Licitra et al. [7]. The feedstuffs were ground to pass through a 1 mm sieve size using a standard laboratory sample mill. Briefly, duplicates of 0.5 g were weighed in 50 mL centrifuge tubes and filled with 40 mL of borate–phosphate buffer (12.20 g NaH_2_PO_4_ × H_2_O + 8.91 g Na_2_B_4_O_7_ × 10 H_2_O/L with pH 6.75).

The effect of an antibiotic solution in SGPM on CP degradation was tested by adding 0.5 mL of Penicillin–Streptomycin solution (10,000 units/mL Penicillin; 10,000 µg/ mL Streptomycin by Thermo Fisher Scientific, Massachusetts, USA). Afterwards, the tubes were placed into a drying oven for 1 h at 39 °C as pre-incubation. After pre-incubation, the SGP solution was added. The SGP solution contained 0.58 U of nonspecific type XIV SGP (Merck KGaA, Darmstadt, Germany) per mL at a ratio of 24 U/g true protein (TP) [19]. The concentration of TP in the samples was calculated according to the Cornell Net Carbohydrate and Protein System (CNCPS) as CP minus non-protein nitrogen (fraction A) [20]. Samples of incubation time 0 h were taken immediately after pre-incubation without the addition of an enzyme solution. Subsequently, the feedstuffs were incubated for 2, 4, 8, 24 and 48 h, respectively. Afterwards, sample tubes were filtered through Whatman #41 filter circles and rinsed out with 100 mL bi-distilled water each. The filters were air-dried overnight, and nitrogen was analyzed in the residues and in blank filters using a FOSS Kjeltec^TM^ 8400 unit (Foss GmbH, Hamburg, Germany). 

**Figure 1:**
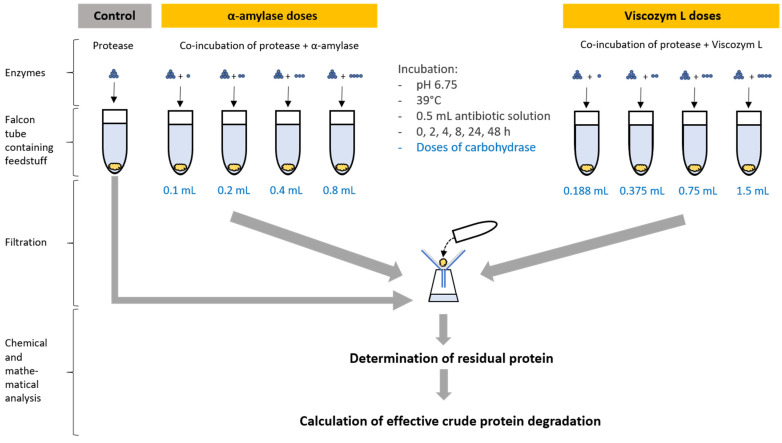
Co-incubation scheme consisting of control variant (*Streptomyces griseus* protease alone) and α-amylase variant and Viscozym^®^ L variant each including four carbohydrase doses.

The effect of carbohydrases in SGPM on CP degradation was investigated by adding fresh α-amylase (Termamyl^®^ 2X, Univar Solutions, Essen, Germany) or Viscozym^®^ L (V2010, Merck KGaA, Darmstadt, Germany) in duplicates, as provided by the manufacturer, after the pre-incubation step. Enzyme specifications and doses are described in Table 1. Subsequently, the SGP solution was added as described above and the SGPM procedure was continued as described above. Figure 1 shows the methodological approach of the co-incubation.

The correction of the co-incubation data for the enzymatical protein of carbohydrase was performed by means of using three runs of blank samples. For this approach, duplicates of falcon tubes containing buffer solution, antibiotic solution and the four aforementioned α-amylase or Viscozym^®^ L doses were incubated for 2, 4, 8, 24 and 48 h and the residual nitrogen was determined as described above.

Concentrations of undegraded protein after SGP incubation (UP) were calculated as follows (considering a sample weight of 0.5 g): UP (g/kg DM) = ((N_residue_ × 6.25 × 10)/(0.5 × DM_feed_)) × 10, where N_resdiue_ is the nitrogen measured in filter residues (mg) corrected by blank filters and the nitrogen of α-amylase or Viscozym^®^ L, and DM_feed_ is the DM concentration of the feedstuff (%). Degraded protein (% of CP) was considered to be the reciprocal of UP at each specific incubation time [24].

In total, the first part of the study consisted of two variants including SGPM with Penicillin–Streptomycin solution and SGPM without Penicillin–Streptomycin solution. Degradation data from the SGPM without added antibiotics were determined prior to the co-incubation approach. The second part of the study comprised 10 variants including CP degradation in sacco, by SGP, by co-incubation of SGP and four α-amylase doses, and by co-incubation of SGP and four Viscozym^®^ L doses. The ED estimates of SGPM without co-incubating carbohydrase (α-amylase/Viscozym^®^ L) were used as the control.

### 2.4. Effective Protein Degradation

The following calculations were made using SAS 9.4 (SAS Institute Inc., Cary, NC, USA). In a first step, the in sacco CP degradation data were corrected for the amount of microbial nitrogen present in the feed residues at each specific incubation time [25]. In a second step, the in sacco CP degradation data of the tested feedstuffs were analyzed by fitting CP degradation (as % of CP) measured after 0, 2, 4, 8, 16, 24, 48 and 72 h of incubation to the exponential Equation (1) provided by McDonald [26] using the MODEL procedure:

DEG = *a* + *b*(1 − e^−*c*(*t* − *L*)^) (1)

where DEG is the disappearance at time *t*, *a* is the washout protein instantly disappearing at time *t* = 0, *b* is the protein potentially degradable in the rumen and *c* is the degradation rate of fraction *b*. The incubation time of corn grain is limited to 24 h incubation as the feedstuff residues almost completely disappeared at 48 h incubation. The possible appearance of a discrete lag phase *L*, at which no ruminal degradation occurs, was considered using a broken-line approach. As long as *t* ≥ *L*, CP degradation was fitted to the regression function, whereas if *t* < *L*, CP degradation was considered to be equal to *a*. The estimates of the lag phase were set to be greater than or equal to zero; *a* + *b* was restricted to be lower than or equal to 100%. The in sacco data set comprised three replicates per feed sample (i.e., three animals).

The in vitro CP degradation was analyzed analogously by the exponential equation provided by McDonald [26] using the MODEL procedure. The in vitro degradation data were corrected for blanks containing specific carbohydrase and antibiotic solutions. Within the in vitro data set, outliers were identified using boxplots and eliminated. Outliers were defined as observations greater than three times the interquartile range. The in vitro data set comprised four replicates (i.e., four runs).

The ED values estimated in sacco and by SGPM were calculated on the basis of the estimated parameters *a*, *b*, *c* and *L* as described by Wulf and Südekum [27] for assumed ruminal passage rates of 0.02 (ED_2_), 0.05 (ED_5_) and 0.08 h^−1^ (ED_8_).

### 2.5. Microscopy

Wheat samples co-incubated with SGP and α-amylase/Viscozym^®^ L, respectively, for a period of 48 h were filtered and air-dried. The feed residue was scraped off the filter and carefully crushed with a spatula within a falcon tube. A small sample amount was stained with 2.5%-Lugol’s iodine (0.25 iodine, 1 g potassium iodide in 1000 mL water) on a microscope slide. After an exposure time of 30 s, a cover slip was placed on the sample and the Lugol’s iodine solution was removed using a paper towel and remoistened using bi-distilled water. The sample was then examined microscopically using an inverted microscope with light as the source (Nikon, Eclipse Ts2, Tokio, Japan) for staining behavior and cell wall conditions at 20× magnification. Microscopy images were produced by a Nikon camera (DS-Fi3, Tokio, Japan) and processed by Nikon imaging software (Element, Software 5.21.01, Tokio, Japan). Microscopy images were used for the descriptive evaluation of the α-amylase and Viscozym^®^ L effects on the enzymatic starch hydrolysis and cell wall conditions.

### 2.6. Chemical Analysis

Concentrations of DM, crude nutrients and detergent fibers were analyzed according to the Association of German Agricultural Analytic and Research Institutes [28] using methods no. 3.1 (DM), no. 4.1.1 (CP kjehldahl), 4.1.2 (CP dumas), 5.1.1 (acid ether extract), 6.1.1 (crude fiber), 6.5.1 (neutral detergent fiber after amylase pre-treatment exclusive of residual ash), 6.5.2 (acid detergent fiber exclusive of residual ash), 7.2.5 (starch) and 8.1 (crude ash), respectively. Starch was determined using the amyloglucosidase method (7.2.5).

Short chain fatty acids produced during fermentation of the silages were determined after aqueous extraction by gas chromatography using a Shimadzu GC2010 (Shimadzu Corp., Kyoto, Japan) fitted with a flame ionization detector described in Okon et al. [15]. Ammonia (NH_3_-N) was determined according to the method of Conway and Byrne [29].

Lactic acid concentrations were determined by a high-performance liquid chromatography device using a Shimadzu LC-20 HPLC fitted with a photo-diode array detector (Shimadzu Corp., Kyoto, Japan), with a 300 mm × 7.8 mm Rezex ROA-Organic Acid H^+^ separation column and a Carbo-H 4 × 3 mm Security Guard cartridge (Phenomenex Ltd., Aschaffenburg, Germany). Silage extracts were mixed 1:1 (*v*/*v*) with 0.016 N sulfuric acid and frozen at −20 °C. Then, thawed extracts were centrifuged 10 min at 14,000 rpm and 20 °C. An amount of 1 mL of the supernatant was mixed with 0.016 N sulfuric acid 1:2 (*v*/*v*) to achieve a total volume of 2 mL. The samples were filtered and injected onto the chromatograph with a volume of 20 μL. The oven temperature was set to 45 °C. An amount of 0.016 N sulfuric acid was used as eluent at 0.6 mL/min isocratic flow. Lactic acid was detected at 210 nm. An amount of 1 g L-(+)-lactic acid in 200 mL 0.016 N sulfuric acid was used as standard stock solution. An 8-point external calibration in a range of 0.2 to 4 g/L was applied.

### 2.7. Statistical Analysis

Statistical analysis was performed using SAS 9.4. Outliers of the in sacco and in vitro estimated ED values were identified by studentized residuals greater than three according to the 3σ rule using PROC UNIVARIATE. Finally, least squares means were estimated for ED at an assumed ruminal passage rate of 2% (ED_2_), 5% (ED_5_) and 8% (ED_8_) per hour separately for each feed using the MIXED procedure and the following model for both hypotheses:

*Y_ij_ = µ + α_i_ + e_ij_* (2)

where *Y_ij_* is ED_2_, ED_5_ and ED_8_; *µ* is the general mean; *α_i_* is the fixed effect of the variant (*i* = 1, 2, where 1 = SGPM with Penicillin–Streptomycin solution, 2 = SGPM without Penicillin–Streptomycin solution and *i* = 1, …, 10, where 1 = the in sacco estimation of CP degradation, 2 = the CP degradation estimated by SGPM, 3 = the CP degradation estimated by the co-incubation of SGP and 0.1 mL α-amylase solution, 4 = the CP degradation estimated by the co-incubation of SGP and 0.2 mL α-amylase solution, 5 = the CP degradation estimated by the co-incubation of SGP and 0.4 mL α-amylase solution, 6 = the CP degradation estimated by the co-incubation of SGP and 0.8 mL α-amylase solution, 7 = the CP degradation estimated by the co-incubation of SGP and 0.188 mL Viscozym^®^ L solution, 8 = the CP degradation estimated by the co-incubation of SGP and 0.375 mL Viscozym^®^ L solution, 9 = the CP degradation estimated by the co-incubation of SGP and 0.750 mL Viscozym^®^ L solution, 10 = the CP degradation estimated by the co-incubation of SGP and 1.5 mL Viscozym^®^ L solution); and *e_ij_* is the random residual effect with *e_ij_*~N(0,σ^2^*e_i_*) or *e_ij_*~N(0,σ^2^*e*). Homogeneous or heterogeneous residual variances were considered according to the likelihood ratio test for the analysis of ED between treatments. Differences between the least squares means with *p* < 0.05 were considered to be significant with the Tukey–Kramer adjustment because the data set is characterized by an unequal number of replications in the treatments. The studentized residuals were confirmed to have Gaussian distribution using the UNIVARIATE procedure.

## 3. Results

The analyzed concentrations of crude nutrients, detergent fibers and starch are presented in Table 2.

All silages were characterized by a low pH and distinct lactic acid concentrations. The concentration of n-butyric acid and NH_3_-N was on a low level and therefore negligible. The concentrations of i-butyric, valeric and caproic acid were below the limit of detection (Table 3).

The ED estimates of all tested feedstuffs were significantly lower when the antibiotic solution was used in SGPM (*p* < 0.05) (Table 3). Regardless of the ruminal passage rate, the largest differences were observed in corn silage (5%-points) by the decreased degradation rate (Table 4 and Table S1).

The in sacco and in vitro estimates of ED are summarized in Table 5. The in sacco ED values were significantly underestimated by SGPM and by SGPM with co-incubated carbohydrase (α-amylase/Viscozym^®^ L) by maximal 60%-points (*p* < 0.05).

When α-amylase and SGP were co-incubated, significantly higher ED was determined in grains of wheat (4–6%-points) and corn (2–3%-points) and in corn silage (3%-points) compared to the control variant (SGPM) (*p* < 0.05).

When Viscozym^®^ L and SGP were co-incubated, higher ED was determined in rapeseed meal (3%-points), wheat grain (11%-points), grass silage (3%-points), PCFPS (3–4%-points) and corn silage (3–6%-points) (*p* < 0.05). The ED of the other feedstuffs was little changed or not affected by the additional carbohydrase compared to the control variant (Table 5). An increase in ED by SGPM with additional carbohydrase was associated with reduced lag time and an increased degradation rate (Tables S2 and S3).

**Figure 2:**
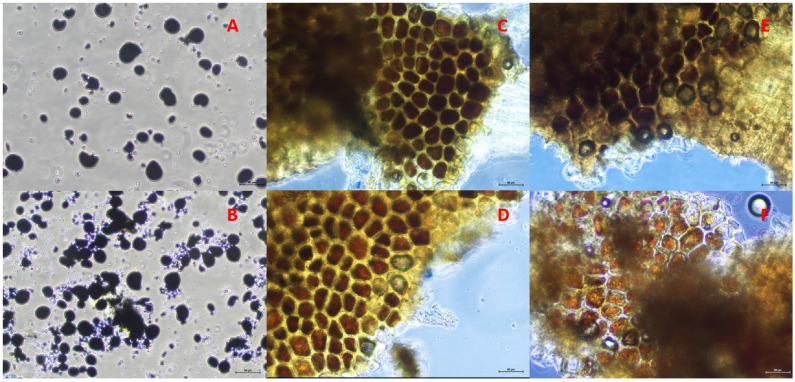
Microscopy images of stained wheat residues with Lugol’s iodine solution after 48 h incubation without (**A**) or with *Streptomyces griseus* protease and α-amylase (**B**–**F**). (**A**): Wheat incubated in borate–phosphate buffer for 48 h; (**B**): Wheat incubated in borate–phosphate buffer with *Streptomyces griseus* protease for 48 h; (**C**): Wheat incubated in borate–phosphate buffer with *Streptomyces griseus* protease + 0.1 mL α-amylase for 48 h; (**D**): Wheat incubated in borate–phosphate buffer with *Streptomyces griseus* protease + 0.2 mL α-amylase for 48 h; (**E**): Wheat incubated in borate–phosphate buffer with *Streptomyces griseus* protease + 0.4 mL α-amylase for 48 h; (**F**): Wheat incubated in borate–phosphate buffer with *Streptomyces griseus* protease + 0.8 mL α-amylase for 48 h. Scale = 50 μm.

**Figure 3:**
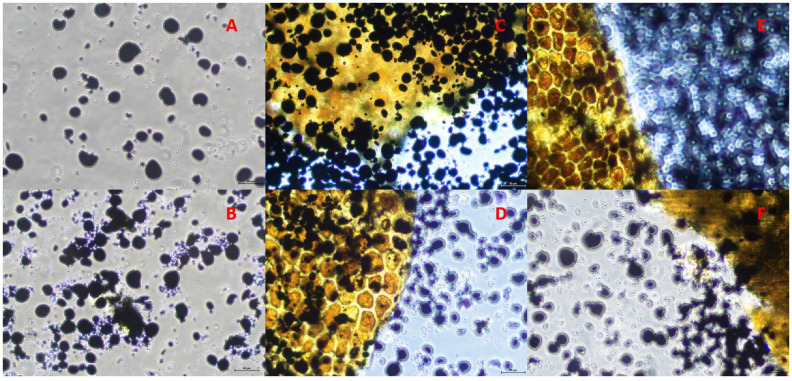
Microscopy images of stained wheat residues with Lugol’s iodine solution after 48 h incubation without (**A**) or with *Streptomyces griseus* protease and Viscozym^®^ L (**B**–**F**). (**A**): Wheat incubated in borate–phosphate buffer for 48 h; (**B**): Wheat incubated in borate–phosphate buffer with *Streptomyces griseus* protease for 48 h; (**C**): Wheat incubated in borate–phosphate buffer with *Streptomyces griseus* protease + 0.188 mL Viscozym^®^ L for 48 h; (**D**): Wheat incubated in borate–phosphate buffer with *Streptomyces griseus* protease + 0.375 mL Viscozym^®^ L for 48 h; (**E**): Wheat incubated in borate–phosphate buffer with *Streptomyces griseus* protease + 0.750 mL Viscozym^®^ L for 48 h; (**F**): Wheat incubated in borate–phosphate buffer with *Streptomyces griseus* protease + 1.5 mL Viscozym^®^ L for 48 h. Scale = 50 μm.

Microscopic images of wheat residues incubated with SGP and α-amylase or Viscozym^®^ L, respectively, for 48 h, showed different staining behaviors depending on the carbohydrase (Figure 2 and Figure 3). Co-incubating SGP and α-amylase resulted in decreased staining intensity as fewer stained starch granules were present. When SGP and Viscozym^®^ L were co-incubated, however, the violet staining was consistently preserved. In every case, the cell wall appeared to stay intact.

## 4. Discussion

The nutrient concentrations of the feedstuffs correspond to the literature data [30,31,32,33,34,35]. The silage fermentation parameters are similar to the literature data, whereby the lactic acid concentrations are higher than those reported in the literature [30,34,35].

The objectives of this study were to investigate the effect of an antibiotic solution in the SGPM and the impact of α-amylase or Viscozym^®^ L co-incubated in the SGPM on ED with reference to in sacco ED. 

The naturally occurring microbes in the feed favor microbial activity during in vitro incubation [36,37]. However, the accuracy of the SGPM is limited by two aspects regarding plant microbes. Similar to rumen microbes, plant-associated microbes could catabolize plant nutrients, i.e., carbohydrates and proteins, for their anabolism during incubation. With respect to the SGP, the plant microbiome could act synergistically with the protease in degrading the CP to a limited extent. The plant microbiome is specific to genotype, plant organ and environmental factors (soil, plant disease, fertilization) [36,37]. The inconsistent microbial composition of incubated plant material could lead to the strongly sample-specific microbial degradation of CP to an unknown extent. The second aspect contributing to the bias of CP degradation data by SGP is microbial mass proliferation during incubation. In particular, the release of fermentable substrates during the co-incubation of SGP and carbohydrase emphasize this effect [12]. Therefore, the use of an antibiotic solution is suggested, enabling uniform incubation conditions [38,39,40,41]. The SGPM protocol of Licitra et al. [7], however, did not recommend the use of an antibiotic solution. The results indicated significantly lower ED estimates with the use of antibiotics. The Penicillin–Streptomycin solution we applied is used in cell cultures to inhibit transpeptidases, which occur exclusively in bacteria [42]. However, the review by Blumberg and Strominger [42] showed that different *Streptomyces* strains secrete enzymes with transpeptidase specificities. It remains unclear whether this specificity also occurs within the SGP mixture, which would explain the reduced ED when using the Penicillin–Streptomycin solution. Nevertheless, preparations that combine different antibiotics appear to be a recommendable tool preventing microbial activity during in vitro incubation and, for reasons of standardization, ensuring reliable estimations of ruminal CP degradation with the SGPM by providing uniform incubation conditions for different feedstuffs.

From a methodological point of view, several aspects can influence in sacco CP degradation estimates. These include microbial attachment, particle losses and animal-related factors; as such, they make reproducible estimates difficult [1]. The SGPM [7] as an alternative method compensates for such limitations by estimating the reproducible ruminal CP degradation under standardized conditions and without the use of animals. However, it was assumed that the feed-specific complexes of protein, starch and fiber act as a physical barrier hindering SGP to access and sufficiently hydrolyze the protein. Therefore, it was proposed to add carbohydrases as co-incubation agents [8,11].

The in sacco ED estimates were used as a reference to evaluate the estimates of ED by SGPM in terms of estimation accuracy. The in sacco ED values of tested feedstuffs were in the range given in the literature [31,33,34]. The in sacco ED values of corn silage and DDGS are slightly higher than those reported in the literature [31,33]. The DDGS is influenced by factors related to its production process (i.e., grinding, heat, drying and pressure) [43] and the corn silage by factors related to growing conditions (i.e., fertilization, maturity, weather conditions) [44]. Consequently, these factors could contribute to the sample-specific degradation properties differing from those in the literature. The ED values estimated by SGPM without carbohydrase were in the range of those in the published literature [8].

The estimates of the in sacco ED were underestimated both by SGPM and by SGPM with carbohydrase (α-amylase/Viscozym^®^ L). Successful co-incubation regarding an improvement in the enzymatic CP degradation was observed with the bromelain protease and α-amylase in cereals [41]. The authors reported an increased enzymatic protein degradation by approximately 8% with the co-incubation of bromelain protease and amylase [41]. However, compared to the SGP mixture of endo- and exopeptidases [45], the bromelain protease is specified by solely endo-protease activity which might be limiting for an efficient CP hydrolysis [46]. Our results revealed slightly increased ED for specific feedstuffs when SGP was co-incubated with α-amylase or Viscozym^®^ L, compared to the control. In wheat grain, an increase of maximal 6 and 11%-points in ED by co-incubating SGP with α-amylase and Viscozym^®^ L was observed, respectively. This effect probably resulted from the synergistic actions of the protease and the carbohydrase affecting the protein matrix that embeds the starch granules and the protein that is enclosed in the starch granules [10,47]. The microscopy images of co-incubated SGP and α-amylase showed discolored cell structures and, macroscopically, no visible wheat starch remained at the bottom of the falcon tubes after 48 h incubation. We concluded that most of the starch was degraded (Figure 2 and Figure S1). Viscozym^®^ L, however, obviously did not degrade the wheat starch during co-incubation (Figure 3 and Figure S2). Co-incubation of SGP and α-amylase seemed to work quite well, but it remains unclear why this was only evident in the wheat grains. Successful co-incubation of protease and carbohydrase was also reported by other studies [10,41,48,49].

Generally, it can be assumed that the carbohydrases did not sufficiently dissolve the feed-specific complexes of proteins and carbohydrates, despite their high doses applied in the SGPM. In particular, the effect of the Viscozym^®^ L on ED estimates was minimal in rapeseed meal, DDGS and the silages, although these feedstuffs are mainly composed of cell wall components such as cellulose, xylose, arabinose and pectin, which are all targets of Viscozym^®^ L [50]. With regard to the α-amylase, its effect was absent in corn grain and in PCFPS, which contained 78 and 20% starch, respectively. The differences between the ED estimated in sacco and that estimated by SGPM with carbohydrase might also be related to the incubation conditions. Enzymatic degradation reactions are generally dependent on a number of factors including the enzyme concentration, incubation time, temperature and pH of the buffer solution [10,51]. The incubation conditions set by the SGPM (39 °C, pH 6.75; [7]) were not the optimum for Viscozym^®^ L and α-amylase. The optimal conditions of Viscozym^®^ L are 44–55 °C in pH 5.0 [23,52] and, according to the manufacturer, for α-amylase are >80 °C in pH 6–7. Therefore, a total of four doses per carbohydrase were used (Table 1). All doses exceeded those recommended by the manufacturer or reported in the literature [9,21], to ensure sufficient enzyme activity and to compensate for inhibitory influences on the conversion of carbohydrate–protein complexes (i.e., unfavorable incubation conditions and the disturbing effect of SGP). The incubation time of 48 h is sufficient for the enzymatic conversion of the substrates contained in the feedstuff since the relevant enzymatic reactions take place in the first hours of incubation [10]. In an experiment, Karimi et al. [10] observed that the degradation of the protein and starch of barley bran mainly occurred within the first three hours considering the optimal temperature range for the protease (Alcalase^®^) and α-amylase (Termamyl^®^). Following this initial phase, higher enzyme activities or longer incubation times did not result in the increased extraction of starch [10]. Another important factor limiting sufficient carbohydrase activity is the assumption that the carbohydrases themselves might act as substrates for the protease during co-incubation [53,54]. Recently published data confirmed the inhibitory effects of SGP on α-amylase or Viscozym^®^ L during co-incubation [11].

## 5. Conclusions

The use of an antibiotic solution slightly reduced in vitro CP degradation. It is recommended to prevent microbial activity and improve the standardization of in vitro estimates. The co-incubation of SGP and carbohydrase did not sufficiently reduce the differences between in sacco and in vitro CP degradation. It seems therefore not to be a promising approach. It is assumed that the incubation conditions and enzyme interactions lead to insufficient activity of the carbohydrases. The pre-incubation of carbohydrase prior to the SGPM appears to be more promising as the carbohydrase requirements for optimal incubation conditions can be implemented. 

## Figures and Tables

**Table 1:** Enzyme density and enzyme activity of α-amylase and Viscozym^®^ L.

	α-Amylase	Viscozym^®^ L
Enzyme activity	240 KNU/g ^1^	≥100 FBGU/g ^2^
Enzyme density	1.25 g/mL ^3^	1.2 g/mL ^4^
Enzyme activity per dose	0.1 mL~30 KNU 0.2 mL~60 KNU 0.4 mL~120 KNU 0.8 mL~240 KNU	0.188 mL~22.56 FBGU 0.375 mL~45 FBGU 0.750 mL~90 FBGU 1.5 mL~180 FBGU
Reference	Cone et al. [9]	Ansharullah et al. [21]

^1^ One kilo novo unit (KNU) is the amount of enzyme that hydrolyzes 4870 mg of starch per hour under standard conditions (pH 5.6, 37 °C and 0.3 mM Ca^2+^) [22]; ^2^ one fungal ß-glucanase unit (FBGU) is the enzyme amount required to hydrolyze barley β-glucan to measurable carbohydrates at standard conditions (pH 5.0, 30 °C, 30 min reaction time) at 1 µmol glucose per min [23]; ^3^ according to the manufacturer (Univar Solutions, Essen, Germany); ^4^ according to the manufacturer (Merck KGaA, Darmstadt, Germany).

**Table 2:** Concentrations of crude nutrients, detergent fibers and starch (g/kg DM).

Feedstuff	DM	CA	CP	AEE	CF	aNDFom	ADFom	Starch
Rapeseed meal	880	75	374	38	156	249	227	n.a.
DDGS	901	47	321	79	75	293	120	n.a.
Wheat grain	984	19	140	24	25	145	34	743
Corn grain	876	14	93	56	27	119	13	783
Grass silage	377	103	151	41	289	500	329	n.a.
PCFPS	610	69	153	22	220	308	274	201
Corn silage	328	38	74	32	188	398	217	443

AEE: acid ether extract; aNDFom: neutral detergent fiber treated with amylase and expressed exclusive of residual ash; ADFom: acid detergent fiber expressed exclusive of residual ash; CA: crude ash; CF: crude fiber; CP: crude protein; DDGS: dried distillers’ grains with solubles; DM: dry matter; n.a.: not analyzed; PCFPS: partial crop field pea silage.

**Table 3:** Ensiling characteristics of the silages.

Feedstuff	DM	pH	Lactic Acid	Acetic Acid	n-Butyric Acid	NH_3_-N
Grass silage	377	4.35	266.8 (14.1)	21.4 (0.0)	1.5 (0.0)	1.8 (0.0)
PCFPS	610	4.38	91.6 (3.5)	13.7 (0.1)	n.d.	1.0 (0.0)
Corn silage	328	3.80	90.9 (1.3)	17.7 (0.3)	n.d.	1.5 (0.0)

DM: dry matter; n.d.: below limit of detection; PCFPS: partial crop field pea silage; DM is given as g /kg; lactic acid, acetic acid, n-butyric acid is given as g/kg DM. NH_3_-N is given as %/CP. Standard deviation is given in brackets.

**Table 4:** Least squares means of effective crude protein degradation (ED, % of CP) at 0.02 (ED_2_), 0.05 (ED_5_) and 0.08 h^−1^ (ED_8_) assumed ruminal passage rates determined by *Streptomyces griseus* protease method with (+) and without antibiotic solution (−).

	ED_2_			ED_5_			ED_8_		
Feedstuff	−	+	*p*-Value	−	+	*p*-Value	−	+	*p-*Value
Rapeseed meal	70 ^a^	69 ^a^	0.1030	62 ^a^	60 ^b^	<0.001	56 ^a^	53 ^b^	<0.001
DDGS	61 ^a^	61 ^a^	0.6266	55 ^a^	55 ^a^	0.0913	51 ^a^	50 ^b^	0.0299
Wheat grains	74 ^a^	71 ^b^	<0.001	71 ^a^	67 ^b^	<0.001	67 ^a^	64 ^b^	<0.001
Corn grains	28 ^a^	25 ^b^	0.0016	27 ^a^	24 ^b^	<0.001	27 ^a^	23 ^b^	<0.001
Grass silage	73 ^a^	70 ^b^	<0.001	71 ^a^	68 ^b^	<0.001	70 ^a^	66 ^b^	<0.001
PCFPS	80 ^a^	80 ^a^	0.5976	79 ^a^	78 ^b^	0.0422	78 ^a^	77 ^b^	0.0412
Corn silage	70 ^a^	65 ^b^	<0.001	69 ^a^	64 ^b^	<0.001	68 ^a^	63 ^b^	<0.001
Range of SE	0.18–0.42			0.16–0.31			0.07–0.32		

^a,b^ different lower-case letters indicate significant differences between ED determined with or without antibiotic solution (*p* < 0.05); DDGS: dried distillers’ grains with solubles; PCFPS: partial crop field pea silage; SE: standard error. The antibiotic solution consisted of 10,000 units/mL Penicillin and 10,000 µg/mL Streptomycin.

**Table 5:** Least squares means of effective crude protein degradation (ED, % of CP) determined in sacco and by co-incubation of *Streptomyces griseus* protease and α-amylase/ Viscozym^®^ L (in vitro) at 0.02 (ED_2_), 0.05 (ED_5_) and 0.08 h^−1^ (ED_8_) assumed ruminal passage rates.

Feedstuff		In Sacco	SGP Solo	SGP + α-A_1_	SGP + α-A_2_	SGP + α-A_3_	SGP + α-A_4_	SGP + V_1_	SGP + V_2_	SGP + V_3_	SGP + V_4_	Range of SE
Rapeseed meal	ED_2_	85 ^A^	69 ^Bb^	70 ^Bb^	69 ^Bb^	69 ^Bb^	69 ^Bb^	71 ^Ba^	71 ^Ba^	72 ^Ba^	72 ^Ba^	0.12–0.84
	ED_5_	78 ^A^	60 ^Bb^	60 ^Bb^	59 ^Bb^	59 ^Bb^	59 ^Bb^	62 ^Ba^	61 ^Ba^	63 ^Ba^	62 ^Ba^	0.28–0.33
	ED_8_	72 ^A^	53 ^Bb^	54 ^Bb^	53 ^Bb^	53 ^Bb^	52 ^Bb^	55 ^Ba^	54 ^Bb^	56 ^Ba^	55 ^Ba^	0.13–1.16
DDGS	ED_2_	93 ^A^	61 ^Bb^	61 ^Bb^	61 ^Bb^	61 ^Bb^	61 ^Bb^	62 ^Ba^	61 ^Bb^	61 ^Bb^	59 ^Bb^	0.08–0.55
	ED_5_	91 ^A^	55 ^Bb^	55 ^Bb^	55 ^Bb^	55 ^Bb^	55 ^Bb^	56 ^Ba^	56 ^Ba^	55 ^Bb^	54 ^Bb^	0.16–0.19
	ED_8_	89 ^A^	50 ^Bb^	50 ^Bb^	51 ^Ba^	50 ^Bb^	51 ^Ba^	51 ^Ba^	52 ^Ba^	51 ^Bb^	50 ^Bb^	0.15–0.17
Wheat grain	ED_2_	94 ^A^	71 ^Bb^	73 ^Ba^	74 ^Ba^	74 ^Ba^	77 ^Ba^	76 ^Ba^	78 ^Ba^	79 ^Ba^	82 ^Ba^	0.16–0.70
	ED_5_	89 ^A^	67 ^Bb^	69 ^Ba^	70 ^Ba^	70 ^Ba^	72 ^Ba^	72 ^Ba^	74 ^Ba^	74 ^Ba^	78 ^Ba^	0.31–0.36
	ED_8_	84 ^A^	64 ^Bb^	66 ^Ba^	67 ^Ba^	67 ^Ba^	68 ^Ba^	68 ^Ba^	70 ^Ba^	71 ^Ba^	75 ^Ba^	0.30–0.34
Corn grain	ED_2_	85 ^A^	25 ^Bb^	27 ^Bb^	27 ^Bb^	27 ^Ba^	28 ^Ba^	27 ^Bb^	25 ^Bb^	25 ^Bb^	25 ^Bb^	0.52–0.60
	ED_5_	74 ^A^	24 ^Bb^	26 ^Bb^	26 ^Ba^	26 ^Ba^	26 ^Ba^	25 ^Ba^	25 ^Ba^	25 ^Bb^	25 ^Ba^	0.10–3.32
	ED_8_	67 ^A^	23 ^Bb^	25 ^Ba^	25 ^Ba^	25 ^Ba^	26 ^Ba^	25 ^Ba^	25 ^Bb^	25 ^Bb^	26 ^Ba^	0.06–3.99
Grass silage	ED_2_	91 ^A^	70 ^Bb^	70 ^Bb^	70 ^Bb^	69 ^Bb^	69 ^Ba^	72 ^Ba^	72 ^Ba^	72 ^Ba^	73 ^Ba^	0.23–0.26
	ED_5_	86 ^A^	68 ^Bb^	68 ^Bb^	68 ^Bb^	67 ^Bb^	67 ^Bb^	69 ^Ba^	69 ^Ba^	69 ^Ba^	68 ^Bb^	0.20–0.23
	ED_8_	83 ^A^	66 ^Bb^	66 ^Bb^	66 ^Bb^	66 ^Bb^	66 ^Bb^	67 ^Ba^	67 ^Ba^	67 ^Ba^	66 ^Bb^	0.21–0.24
Partial crop field pea silage	ED_2_	90 ^A^	80 ^Bb^	80 ^Bb^	80 ^Bb^	80 ^Bb^	80 ^Bb^	83 ^Ba^	83 ^Ba^	83 ^Ba^	83 ^Ba^	0.25–0.29
	ED_5_	87 ^A^	78 ^Bb^	79 ^Bb^	79 ^Bb^	78 ^Bb^	79 ^Bb^	81 ^Ba^	81 ^Ba^	81 ^Ba^	82 ^Ba^	0.16–0.19
	ED_8_	84 ^A^	77 ^Bb^	77 ^Bb^	77 ^Bb^	77 ^Bb^	78 ^Bb^	79 ^Ba^	80 ^Ba^	80 ^Ba^	80 ^Ba^	0.18–0.21
Corn silage	ED_2_	92 ^A^	65 ^Bb^	68 ^Ba^	67 ^Bb^	67 ^Bb^	68 ^Ba^	69 ^Ba^	69 ^Ba^	69 ^Ba^	71 ^Ba^	0.38–0.44
	ED_5_	89 ^A^	64 ^Bb^	66 ^Ba^	66 ^Ba^	66 ^Ba^	67 ^Ba^	67 ^Ba^	67 ^Ba^	67 ^Ba^	66 ^Ba^	0.26–0.30
	ED_8_	86 ^A^	63 ^Bb^	65 ^Ba^	65 ^Ba^	65 ^Ba^	66 ^Ba^	66 ^Ba^	65 ^Ba^	66 ^Ba^	65 ^Ba^	0.25–0.28

^A,B^ different upper-case letters indicate significant differences between ED values estimated in sacco and in vitro, respectively (*p <* 0.05). ^a,b^ different lower-case letters indicate significant differences between ED values estimated by SGP solo and by SGP + α-A_n_/ SGP + V_n_ (*p <* 0.05). α-A: α-amylase; CP: crude protein; DDGS: dried distillers’ grains with solubles; SGP: *Streptomyces griseus* protease; SE: standard error; V: Viscozym^®^ L; SGP + α-A_1_: co-incubation of SGP and 0.1 mL α-amylase; SGP + α-A_2_: co-incubation of SGP and 0.2 mL α-amylase; SGP + α-A_3_: co-incubation of SGP and 0.4 mL α-amylase; SGP + α-A_4_: co-incubation of SGP and 0.8 mL α-amylase; SGP + V_1_: co-incubation of SGP and 0.188 mL Viscozym^®^ L; SGP + V_2_: co-incubation of SGP and 0.375 mL Viscozym^®^ L; SGP + V_3_: co-incubation of SGP and 0.750 mL Viscozym^®^ L; SGP + V_4_: co-incubation of SGP and 1.5 mL Viscozym^®^ L. The in sacco degradation data were corrected for microbial nitrogen according to Parand and Spek [25]. The degradation data estimated by enzymatic co-incubation were corrected for the enzymatic protein of α-amylase/ Viscozym^®^ L.

## Data Availability

The data presented in this study are available on request from the corresponding author.

## Supplementary Materials

The following supporting information can be downloaded at: https://www.mdpi.com/article/10.3390/ani14202982/s1, Table S1: Means of estimated parameters of in vitro crude protein degradation determined by *Streptomyces griseus* protease method with (+) and without antibiotic solution (−); Table S2: Means of estimated parameters of protein degradation determined in sacco and by co-incubation of *Streptomyces griseus* protease and α-amylase; Table S3: Means of estimated parameters of protein degradation determined in sacco and by co-incubation of *Streptomyces griseus* protease and Viscozym^®^ L; Figure S1: Falcon tubes containing wheat incubated 48 h with *Streptomyces griseus* protease and co-incubated with *Streptomyces griseus* protease and α-amylase; Figure S2: Falcon tubes containing wheat incubated 48 h with *Streptomyces griseus* protease and co-incubated with *Streptomyces griseus* protease and Viscozym^®^ L.
