# Supplementary material for: Ruminal Crude Protein Degradation Determined in Sacco and by Co-Incubation of Streptomyces griseus Protease and Carbohydrases
Source: Animals (Basel). 2024 Oct 16;14(20):2982. doi: 10.3390/ani14202982 (PMC11506112; doi:10.3390/ani14202982)
Supplement: Supplementary file 1 [file animals-14-02982-s001.zip › animals-3231103-supplementary.pdf]

Supplements

Table S1: Means of estimated parameters of *in vitro* crude protein degradation determined by *Streptomyces griseus* protease method with (+) and without antibiotic solution (-)

	Without antibiotic solution				With antibiotic solution			
	a	*b*	*c*	*L*	*a*	*b*	*c*	*L*
Rapeseed meal	23	55	0.12	0.1	21	57	0.11	0.5
DDGS	27	38	0.14	0	27	40	0.12	0
Wheat	34	44	0.27	0	40	33	4.59	3.8
Corn grain	26	2	0.08	4.0	22	12	0.01	0
Grass silage	64	11	0.09	0.8	61	13	0.05	0.2
PCFPS	72	10	0.13	0	71	10	0.10	0
Corn silage	65	6	0.12	0	60	8	0.05	0

a: washout protein, which is instantly disappearing; *b*: potentially degradable protein; *c*: rate of crude protein degradation; DDGS: dried distillers' grains with solubles; *L*: lag time; PCFPS: partial crop field pea silage

a and *b* are given as %; *c* is given as % h^{-1}; *L* is given as h.

The antibiotic solution consisted of 10,000 units/mL Penicillin and 10,000 µg/ mL Streptomycin.

Table S2: Means of estimated parameters of protein degradation determined *in sacco* and by co-incubation of *Streptomyces griseus* protease and α-amylase

	In sacco				SGP + α-A_1				SGP + α-A_2				SGP + α-A_3				SGP + α-A_4			
	a	*b*	*c*	*L*	*a*	*b*	*c*	*L*	*a*	*b*	*c*	*L*	*a*	*b*	*c*	*L*	*a*	*b*	*c*	*L*
Rapeseed meal	37	51	4.43	3.9	21	58	0.10	0	21	58	0.10	0.2	21	58	0.10	0	21	58	0.10	0
DDGS	76	20	0.15	0	27	41	0.11	0	28	39	0.12	0	27	40	0.12	0	61	10	0.06	0
Wheat	48	49	4.25	3.7	40	36	4.64	3.8	40	36	4.54	3.8	40	37	4.64	3.9	32	49	0.22	0
Corn grain	39	61	0.07	0	23	7	0.03	0	23	5	0.06	2.8	23	7	0.03	0	23	5	0.08	0
Grass silage	61	34	0.14	0	61	12	0.06	0	61	12	0.06	0	61	11	0.06	0	61	10	0.06	0
PCFPS	66	26	4.52	3.8	71	11	0.11	0	71	11	0.12	0	71	11	0.11	0	71	11	0.13	0
Corn silage	77	18	0.08	0	61	9	0.08	0	60	9	0.10	0	60	8	0.16	0	60	9	0.13	0

a: washout protein, which is instantly disappearing; *b*: potentially degradable protein; *c*: rate of crude protein degradation; DDGS: dried distillers' grains with solubles; *L*: lag time; PCFPS: partial crop field pea; SGP: *Streptomyces griseus* protease; SGP + α-A_1: co-incubation of SGP and 0.1 mL α-amylase; SGP + α-A_2: co-incubation of SGP and 0.2 mL α-amylase; SGP + α-A_3: co-incubation of SGP and 0.4 mL α-amylase; SGP + α-A_4: co-incubation of SGP and 0.8 mL α-amylase.

a and *b* are given as %; *c* is given as % h^{-1}; *L* is given as h.

The *in sacco* degradation data was corrected for microbial nitrogen according to Parand and Spek (2021).
The degradation data from co-incubation were corrected for enzymatic protein of α-amylase.

Table S3: Means of estimated parameters of protein degradation determined *in sacco* and by co-incubation of *Streptomyces griseus* protease and Viscozym® L

	SGP + V_1				SGP + V_2				SGP + V_3				SGP + V_4			
	a	*b*	*c*	*L*	*a*	*b*	*c*	*L*	*a*	*b*	*c*	*L*	*a*	*b*	*c*	*L*
Rapeseed meal	21	59	0.11	0	21	60	0.10	0.3	21	61	0.11	0	21	61	0.10	0
DDGS	27	41	0.12	0	27	39	0.13	0	27	39	0.12	0	27	36	0.14	0
Wheat	32	49	0.23	0	32	49	0.26	0	32	51	0.25	0	32	53	0.31	0
Corn grain	23	6	0.02	0	23	3	0.10	0	24	76	0.24	i.e.	21	4	i.e.	0
Grass silage	61	14	0.06	0	61	15	0.06	0	60	15	0.06	0	62	22	0.02	0
PCFPS	71	14	0.10	0	73	10	4.07	3.9	73	10	4.30	3.9	74	9	4.58	3.8
Corn silage	60	11	0.08	0	60	11	0.07	0	60	11	0.08	0	62	38	0.01	0

a: washout protein, which is instantly disappearing; *b*: potentially degradable protein; *c*: rate of crude protein degradation; DDGS: dried distillers' grains with solubles; i.e.: implausible estimate; *L*: lag time; PCFPS: partial crop field pea silage; SGP: *Streptomyces griseus* protease; SGP + V1: co-incubation of SGP and 0.188 mL Viscozym® L; SGP + V2: co-incubation of SGP and 0.375 mL Viscozym® L; SGP + V3: co-incubation of SGP and 0.750 mL Viscozym® L; SGP + V4: co-incubation of SGP and 1.5 mL Viscozym® L.
The degradation data from co-incubation were corrected for enzymatic protein of Viscozym® L.

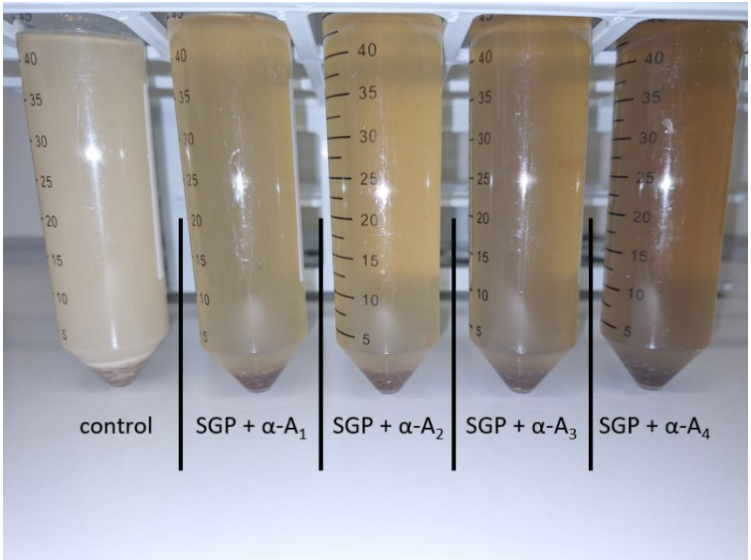

SGP: *Streptomyces griseus* protease; α-A_1- α-A_4: α-amylase doses
Control: incubation of wheat with *Streptomyces griseus* protease
SGP + α-A_1: co-incubation of wheat with SGP and 0.1 mL α-amylase
SGP + α-A_2: co-incubation of wheat with SGP and 0.2 mL α-amylase
SGP + α-A_3: co-incubation of wheat with SGP and 0.4 mL α-amylase
SGP + α-A_4: co-incubation of wheat with SGP and 0.8 mL α-amylase
Co-incubation of SGP and α-A resulted in no visible wheat starch remaining at the bottom of the falcon tubes after 48 h incubation, indicating α-A activity during incubation.

Figure S1: Falcon tubes containing wheat incubated 48 h with *Streptomyces griseus* protease and co-incubated with *Streptomyces griseus* protease and α-amylase

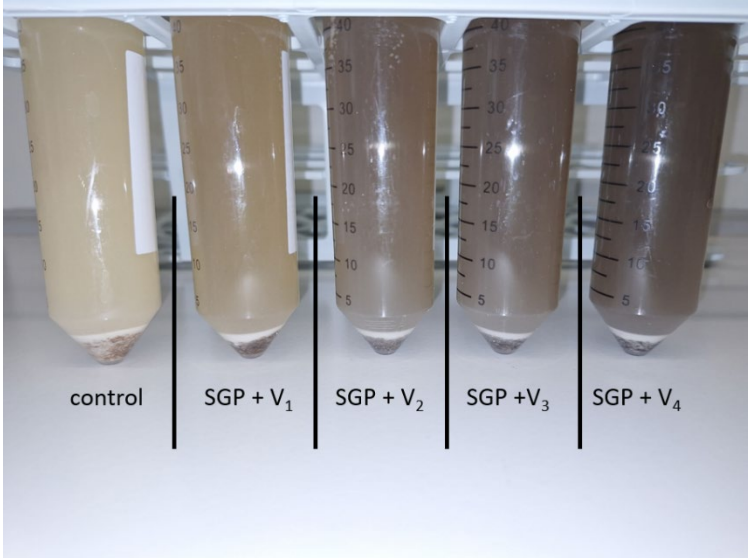

SGP: *Streptomyces griseus* protease; V_1 - V_4: Viscozym® L doses
Control: incubation of wheat with *Streptomyces griseus* protease
SGP + V_1: co-incubation of wheat with SGP and 0.188 mL Viscozym® L
SGP + V_2: co-incubation of wheat with SGP and 0.375 mL Viscozym® L
SGP + V_3: co-incubation of wheat with SGP and 0.750 mL Viscozym® L
SGP + V_4: co-incubation of wheat with SGP and 1.5 mL Viscozym® L
Co-incubation of SGP and V resulted in visible wheat starch remaining at the bottom of the falcon tubes after 48 h incubation, indicating no V activity during incubation.

Figure S2: Falcon tubes containing wheat incubated 48 h with *Streptomyces griseus* protease and co-incubated with *Streptomyces griseus* protease and Viscozym® L
